# Preventative effects of the partial RANKL peptide MHP1-AcN in a mouse model of imiquimod-induced psoriasis

**DOI:** 10.1038/s41598-019-51681-0

**Published:** 2019-10-28

**Authors:** Nan Ju, Munehisa Shimamura, Hiroki Hayashi, Yuka Ikeda, Shota Yoshida, Ayumi Nakamura, Ryuichi Morishita, Hiromi Rakugi, Hironori Nakagami

**Affiliations:** 1 Department of Health Development and Medicine, Osaka University Graduate School of Medicine, Osaka, Japan; 2 Department of Geriatric and General Medicine, Osaka University Graduate School of Medicine, Osaka, Japan; 3 Department of Neurology, Osaka University Graduate School of Medicine, Osaka, Japan; 4 Department of Pharmacy and Department of Medical Innovation, Osaka University Hospital, Osaka, Japan; 5 Department of Clinical Gene Therapy, Osaka University Graduate School of Medicine, Osaka, Japan

**Keywords:** Target identification, Psoriasis

## Abstract

We recently developed a partial peptide of receptor activator of nuclear factor-кB ligand (RANKL) known as microglial healing peptide 1 (MHP1-AcN), that inhibits Toll-like receptor (TLR)-related inflammation through RANKL/RANK signaling in microglia and macrophages without promoting osteoclast activation. The abnormal activation of TLRs contributes to the initiation and maintenance of psoriasis, which is a chronic inflammatory skin disease that involves the aberrant expression of proinflammatory cytokines and the subsequent dermal γδ T cell and T helper 17 (Th17) cell responses. The inhibition of TLR-mediated inflammation provides an important strategy to treat psoriasis. Here, we examined the preventative effects of MHP1-AcN in a mouse model of imiquimod (a TLR 7/8 agonist)-induced psoriasis. Topical imiquimod application induced psoriasis-like skin lesions on the ear and dorsal skin. Systemic administration of MHP1-AcN by daily subcutaneous injection significantly prevented the development of skin lesions, including erythema, scaling and thickening. Mice treated with MHP1-AcN showed reduced levels of skin *Il6* mRNA at 32 h and reduced levels of *Il23* and *Il17a* mRNA at d9. Serum levels of IL-6 and IL-23 were reduced at 32 h, and IL-17A was reduced at d9. These results indicated that MHP1-AcN could decrease imiquimod-induced IL-6, IL-23 and IL-17A production. MHP1-AcN is potentially an alternative treatment for psoriasis.

## Introduction

Psoriasis is one of the most common immune-mediated disorders, and crosstalk between components of the innate and adaptive immune systems greatly contributes to its pathogenesis^1^. Imiquimod (IMQ), a Toll-like receptor (TLR) 7/8 agonist, exacerbates psoriasis in patients and causes psoriasis-like dermatitis in mice^2^. TLR activation triggers macrophages and dendritic cells to produce IL-23, IL-1β and other proinflammatory cytokines, including IL-6 and TNF-α^3^. IL-6 has been associated with macrophage activation, Th17 cell differentiation, and inhibition of regulatory T cell (Treg) activity during the development of psoriasis^4,5^. IL-23 is predominantly secreted by dendritic cells and macrophages to stimulate dermal γδ T cell activation and expansion and positively regulate differentiation or effector function of Th17 cells^6,7^. Dermal γδ T cells have been demonstrated to be the major IL-17 producers in mouse skin^8^. IL-17 acts on keratinocytes to induce hyperproliferation, contributing to the pathogenesis of psoriasis^9,10^. Additionally, antagonists of TLR7/8/9 have been demonstrated to inhibit Th1 and Th17 responses in a model of IL-23-induced psoriasis^11^. Thus, inhibition of TLR-mediated inflammation may provide an effective treatment strategy for psoriasis. Receptor activator of nuclear factor-кB ligand (RANKL) is one such candidate molecule since it can inhibit TLR-mediated inflammation in activated macrophages^12^ and microglia^13^. Additionally, a recent report showed that epidermal RANKL/RANK signaling changes dendritic cell function to maintain the number of peripheral Tregs and suppress allergic contact hypersensitivity responses and the development of systemic autoimmunity^14^. In addition, the developmental pathways of Th17 cells and Tregs are reciprocally regulated and influence the outcome of autoimmune and inflammatory diseases, including psoriasis. Thus, we speculated that RANKL/RANK signaling may improve psoriasis by inhibiting TLR-mediated inflammation and consequently decrease IL-17A production by dermal γδ T cells.

However, systemic administration of recombinant RANKL induces osteoporosis^15^, which is a problem for clinical applications. To solve this problem, we developed a novel peptide, microglia healing peptide-1 (MHP1), which is a partial RANKL peptide. This peptide was shown to inhibit TLR2-, 4-, and 7/8-related inflammation through the RANK signaling pathway without inducing osteoclast differentiation^16,17^. The peptide includes the DE loop and part of the EF loop, which is the binding site of RANKL for its receptor, RANK, but it does not include the AA’ and CD loops that are responsible for osteoclastogenesis. We further modified MHP1 with N-terminal acetylation and C-terminal amidation (MHP1-AcN), which increased its stability and strengthened its inhibitory effects on TLR-mediated inflammation in microglia/macrophages^18^. Systemic administration of MHP1 and MHP1-AcN has been shown to be effective for the treatment of ischemic stroke in mice^16,18^. Therefore, we hypothesized that this peptide may be a novel agent for the treatment of psoriasis.

Here, we examined the preventative effects of different doses of MHP1-AcN on psoriatic skin lesions and IL-17A production and determined the optimum dose in a mouse model of IMQ-induced psoriasis. The influence of MHP1-AcN on the IMQ-induced cytokines IL-6 and IL-23 in mouse skin and serum was assessed. Moreover, we examined the inhibitory effects of MHP1-AcN on IL-6 production in the R837 (TLR7 agonist)-stimulated macrophage cell line.

## Results

### Systemic administration of MHP1-AcN inhibited the development of IMQ-induced psoriasis-like skin lesions and IL-17A production

**Figure 1:**
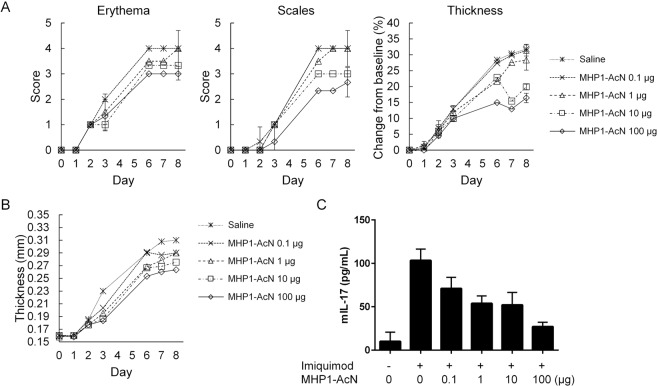
Systemic administration of MHP1-AcN inhibited the development of IMQ-induced skin lesions and IL-17A production. MHP1-AcN (0.1, 1, 10, or 100 µg/mouse) or saline was systemically administered by daily subcutaneous injection at a distant site from IMQ application. MHP1-AcN was injected immediately before IMQ application. (**A**) Dorsal skin erythema and scales were recorded and measured on a four-point scale (0 = none; 1 = slight; 2 = moderate; 3 = marked; and 4 = very marked). Dorsal skin thickness was measured using a digital caliper and calculated as the percentage change from baseline. (**B**) Right ear thickness from day 0–8. (**C**) Mice were sacrificed on d8, and serum IL-17A levels were measured by ELISA. N = 3 per normal group; N = 4 per saline-treated IMQ group; N = 4 per MHP1-AcN-treated IMQ group. All values are expressed as the mean with SEM.

IMQ cream was applied to the shaved dorsal skin and right ears of BALB/c mice for 8 consecutive days. MHP1-AcN (0.1, 1, 10, 100 µg/mouse) or saline was systemically administered by daily subcutaneous injection at a distant site from IMQ application. Dorsal skin erythema and scales were measured on a four-point scale. Dorsal skin thickness was measured and calculated as the percentage change from baseline (Fig. 1A). Ear thickness was recorded on days 0–8 (Fig. 1B). Higher doses of MHP1-AcN resulted in reduced erythema and scale scores and less dorsal skin and ear thickening. However, a higher dose of MHP1-AcN (250 µg/mouse) did not further inhibit the development of IMQ-induced skin lesions (Supplementary Fig. 1). Because topical application of IMQ triggers TLR-mediated inflammation that activates dermal γδ T cells to produce IL-17A^2^, which plays a critical role in the pathogenesis of psoriasis, we next examined serum IL-17A levels in mice. Higher doses of MHP1-AcN showed a tendency to reduce serum IL-17A production without significant difference (Fig. 1C), whereas a much higher dose of MHP1-AcN (250 µg/mouse) did not further reduce IL-17A production (Supplementary Fig. 1). Therefore, we determined the optimum dose of MHP1-AcN (100 µg/mouse) for psoriasis treatment.

### MHP1-AcN inhibited IMQ-induced proinflammatory cytokine expression

**Figure 2:**
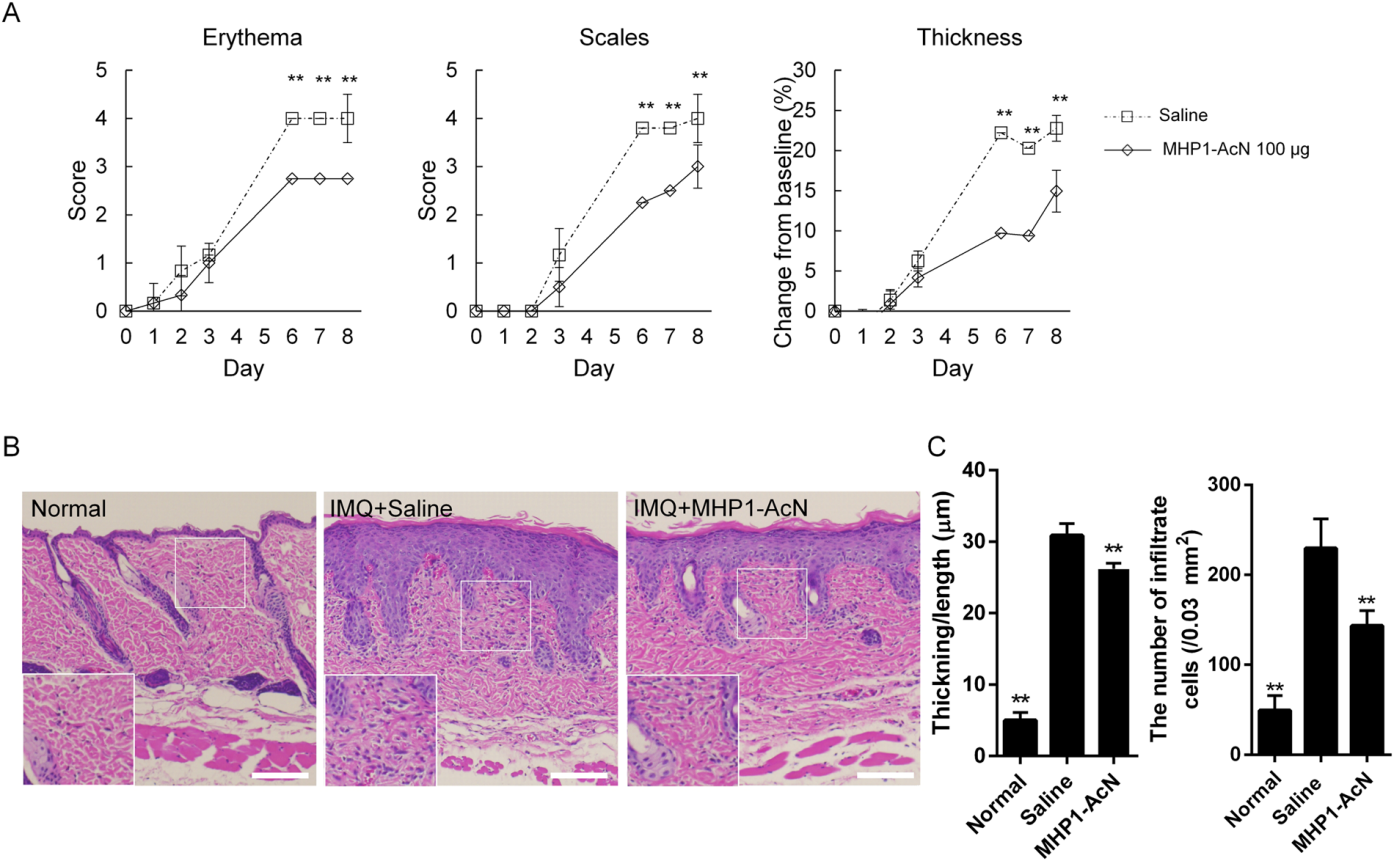
The optimum dose of MHP1-AcN inhibited the development of IMQ-induced skin lesions and inflammatory infiltrates in skin. MHP1-AcN (100 µg/mouse) or saline was systemically administered by daily subcutaneous injection at a distant site from IMQ application. (**A**) Dorsal skin erythema and scales were recorded and measured on a four-point scale (0 = none; 1 = slight; 2 = moderate; 3 = marked; and 4 = very marked). Dorsal skin thickness was measured using a digital caliper and calculated as the percentage change from baseline. (**B**) Representative H&E-stained section of dorsal skin. Scale bar: 100 μm. The white rectangular area in the left lower corner in each staining shows a magnified image of the corresponding area. (**C**) Thickness was calculated as the area of epidermis (µm^2^)/the length of the basement membrane (µm) in more than 10 fields. Inflammatory infiltrate in the dermis was quantified and shown as the number of infiltrated cells/(0.03 mm^2^). **P < 0.01 vs. the saline-treated IMQ group. N = 3 per normal group; N = 4 per saline-treated IMQ group; N = 4 per MHP1-AcN-treated IMQ group. All values are expressed as the mean with SEM.

We next examined IMQ-induced skin inflammation. MHP1-AcN (100 µg/mouse) or saline was systemically administered by daily subcutaneous injection at a distant site from IMQ application. As expected, MHP1-AcN inhibited the development of IMQ-induced skin erythema, scaling, and thickening (Fig. 2A). Hematoxylin and eosin (H&E)-stained IMQ-stimulated dorsal skin was characterized by increased epidermal thickening, parakeratosis, elongated rete ridges, and a mixed cellular infiltrate compared to normal skin (Fig. 2B). Thickening was calculated as the area of epidermis (µm^2^)/the length of the basement membrane (µm), and the inflammatory infiltrate was quantified as the number of infiltrated cells/(0.03 mm^2^). MHP1-AcN treatment decreased epidermal thickening and reduced inflammatory infiltrates compared to IMQ with saline-treated mice (Fig. 2B,C). These results suggest that MHP1-AcN inhibited IMQ-induced skin inflammation.

Proinflammatory cytokines secreted by macrophages and dendritic cells contribute to the pathogenesis of psoriasis. Among these cytokines, IL-6 has been reported to be present at high levels in psoriatic skin and in the plasma of psoriasis patients, suggesting its critical role in psoriasis^19^. In addition to IL-6, a transient increase in IL-23 followed by IL-17A production was observed in the IMQ-induced psoriasis model^2^. Moreover, IMQ-induced dermatitis was almost completely blocked in IL-23- or IL-17RA-deficient mice^2^, proving the pivotal role of the IL-23/IL-17 axis in the pathogenesis of psoriasis^9^. In another IMQ-induced psoriasis study, IMQ-induced *Il6* and *Il23* mRNAs were highly expressed on day 2, whereas *Il17a* was highly expressed on day 4^20^. Thus, we examined IL-6, IL-23, and IL-17A expression at 32 h (early stage) and d9 (late stage).

**Figure 3:**
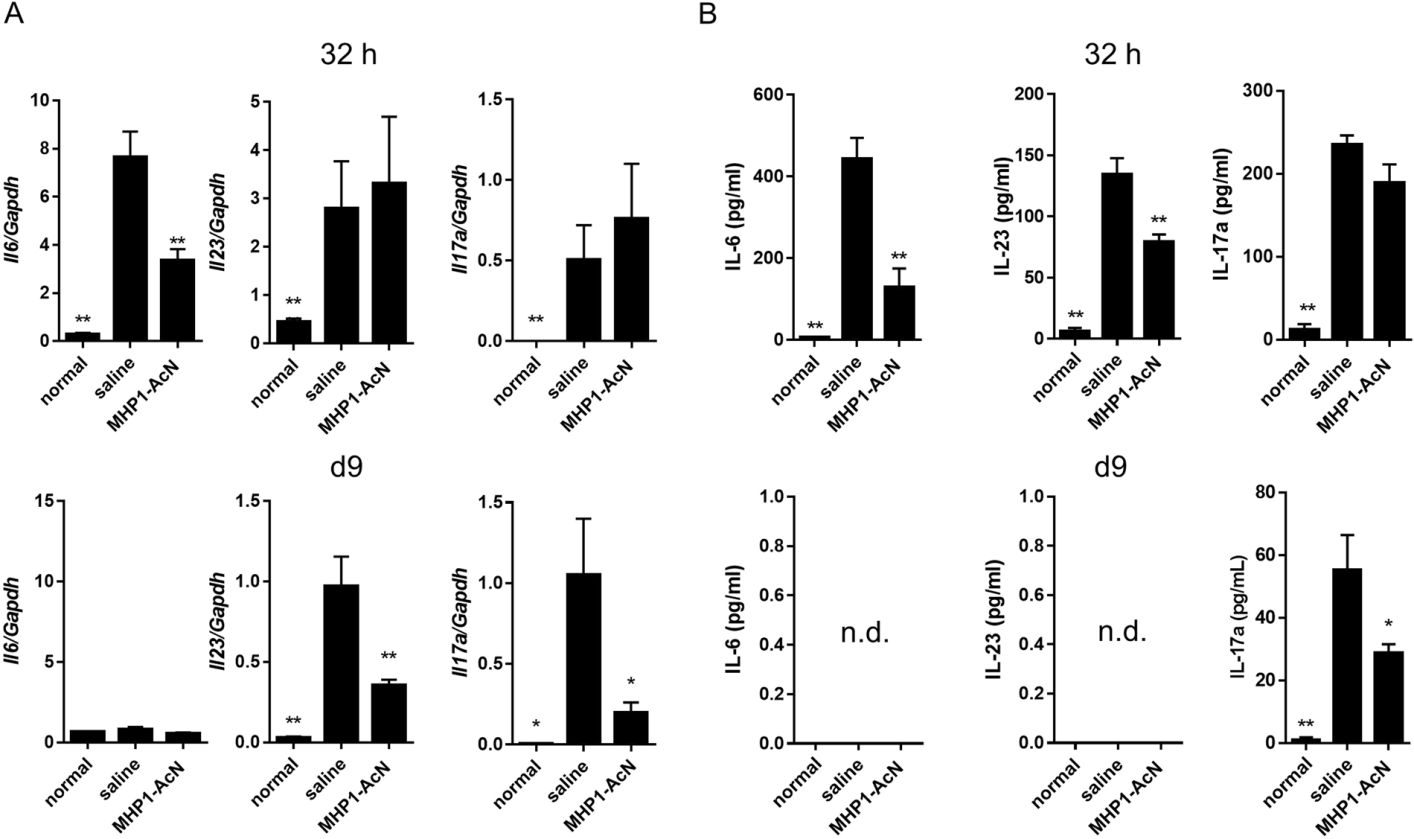
Systemic administration of MHP1-AcN inhibited IMQ-induced IL-6, IL-23 and IL-17A expression. MHP1-AcN (100 µg/mouse) or saline was systemically administered by daily subcutaneous injection at a distant site from IMQ application. Mice were sacrificed at 32 h or d9 in two independent experiments. Dorsal skin samples where IMQ was applied and serum samples were collected. (**A**) The mRNA expression of *Il6*, *Il23*, and *Il17a* at 32 h and d9 in dorsal skin was analyzed by real-time PCR. (**B**) Serum IL-6, IL-23, and IL-17A levels at 32 h and d9 were measured by ELISA. *P < *0.05*, **P < 0.01 vs. the saline-treated IMQ group. 32 h: N = 3 per normal group; N = 4 per saline-treated IMQ group; N = 4 per MHP1-AcN-treated IMQ group, d9: N = 3 per normal group; N = 4 per saline-treated IMQ group; N = 4 per MHP1-AcN-treated IMQ group. All values are expressed as the mean with SEM.

Systemic administration of MHP1-AcN inhibited IMQ-induced skin *Il6* mRNA expression at 32 h, but the result at d9 was not significant. Interestingly, skin *Il23* and *Il17a* mRNA expression was inhibited at d9, but not at 32 h (Fig. 3A). In serum, MHP1-AcN inhibited IMQ-induced IL-6 and IL-23 production at 32 h, whereas IL-17A was only inhibited at d9 (Fig. 3B). IL-6 or IL-23 was not detected at d9.

### MHP1-AcN inhibited R837 (TLR7 agonist)-induced IL-6 production in RAW 264.7 cells

**Figure 4:**
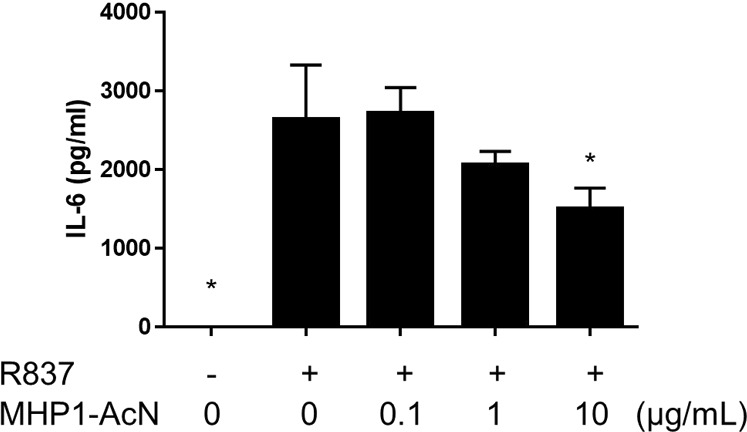
MHP1-AcN inhibited R837-induced IL-6 production in RAW 264.7 cells. RAW 264.7 cells were stimulated with R837 (1 µg/mL) in the presence or absence of MHP1-AcN (0.1, 1, or 10 µg/mL) at the same time for 24 h. Cultured medium was collected and analyzed for IL-6 production by ELISA. *P < *0.05* vs. the group treated with R837 without MHP1-AcN. N = 4 per group. All values are expressed as the mean with SEM.

We previously showed that MHP1 inhibited R848 (an analog TLR7/8 agonist)-induced IL-6 production in microglial cells^16^, and MHP1-AcN significantly inhibited lipopolysaccharide (LPS)-induced IL-6 at concentrations of 1 and 10 µg/mL^18^. RAW 264.7 cells were treated with 1 µg/mL of R837 (a TLR7 agonist) and MHP1-AcN (0.1, 1, 10 µg/mL) at the same time and incubated for 24 h, and supernatants were collected and assayed for IL-6 production. MHP1-AcN inhibited R837-induced IL-6 production in RAW 264.7 cells at a concentration of 10 µg/mL (Fig. 4).

## Discussion

In this study, we demonstrated that the novel, modified peptide MHP1-AcN, which was structurally designed from RANKL and modified with N-terminal acetylation and C-terminal amidation to improve its stability and effectiveness, significantly prevented the development of IMQ-induced psoriasis in mice.

Previous studies showed that MHP1-AcN is a partial agonist of RANK, and it can decrease the TLR2-, TLR4-, and TLR7/8-induced inflammatory cytokines in the microglial cell line MG6, as well as the TLR4-induced inflammatory cytokines in the macrophage cell line RAW264.7^16,17^. In psoriasis, IL-6 and IL-23 are proinflammatory cytokines secreted by activated dendritic cells and macrophages in response to pathogen components or damage-associated molecular patterns (DAMPs) via TLRs and are able to induce dermal γδ T cell activation and expansion and Th17 cell differentiation, contributing to the initiation and maintenance of psoriasis^4,8,21^. In the present study, MHP1-AcN was shown to inhibit TLR7/8 agonist-induced IL-6 production in RAW 264.7 cells and in a mouse model of psoriasis at the early stage of disease. Compared to a recent study showing that an approximately 40% reduction in IL-6 in mice treated with cycloastragenol resulted in better clinical outcomes^22^, a 71.1% decrease in serum IL-6 by MHP1- AcN at the early stage might be enough to be associated with better clinical outcomes. There is evidence supporting the beneficial effects of IL-6 inhibition in the treatment of psoriasis. For example, psoriasis-like skin inflammation induced by intradermal injection of recombinant IL-23 is abrogated in IL-6 knockout mice^23^. However, the IL-6 blockade strategy shows few consistent beneficial effects when used to treat plaque-type psoriasis and psoriatic arthritis (PsA). For example, the humanized anti-IL-6 receptor monoclonal antibody, tocilizumab (Actemra), and the humanized anti-IL-6 monoclonal antibody, clazakizumab, do not improve psoriatic skin lesions^24–27^, and in fact, they even exacerbate the lesions in some patients^26,28^. A possible reason for these findings is that the therapeutic blockade of IL-6 may lead to overcompensation by other proinflammatory cytokines in the skin, which may abrogate the benefits of anti-IL-6 agents^5,29^. In contrast to antibodies against IL-6 or the IL-6 receptor, MHP1-AcN inhibited not only the expression of IL-6 but also IL-23, which emerged as an attractive therapeutic target for moderate-to severe psoriasis by using anti-IL-23 antibodies, such as guselkumab, tildrakizumab and risankizumab^7^. Although the direct action of MHP1-AcN on IL-23 and its effects in the IL-23-induced psoriasis model need further studies, one of the preventative effects of MHP1-AcN on psoriasis may be associated with the inhibition of TLR7/8-induced IL-6 and IL-23 expression.

Dermal γδ T cells are the main source of IL-17 upon IL-23 stimulation, which is predominantly secreted by skin dendritic cells and macrophages in the IMQ-induced psoriasis model^8^. Therefore, limited IL-17A production is one of the consequences of restricted macrophage activation by MHP1-AcN treatment in the present study. The action of MHP1-AcN in dendritic cells and other mechanisms regulating γδ T cell responses, such as the expression of CD69, BTLA, and PD-1, need further study.

Although IMQ-induced psoriasis recapitulates limited aspects of human psoriasis and is highly dependent on a prevalent population of dermal γδ T cells, which is a minor population in human skin^30^, the activation of TLR7/8 in human dendritic cells was reported to contribute to psoriasis^31^, and TLR7/8/9 antagonists reduced moderate-to-severe plaque psoriasis in a phase IIa trial^32^. Based on the aforementioned evidence, MHP1-AcN may also be effective for human psoriasis.

Considering that the plasma half-life of intravenously injected MHP1-AcN has been reported to be less than 10 min^18^, we speculate that the half-life of subcutaneously injected MHP1-AcN will also be short. However, the therapeutic effects of MHP1-AcN were demonstrated after a single subcutaneous injection each day. This is probably because some of the degradation products in the plasma include key sequences needed for anti-inflammatory effects^16^ and these key sequences may still be able to affect immune cells. A similar effect has been reported for glatiramer acetate, which is a copolymer of four amino acids with specific stoichiometry and ranging in length from 40 to 90 amino acids. Glatiramer acetate has shown efficacy in treating multiple sclerosis, despite its very short half-life^33^.

Psoriasis usually precedes PsA onset by an average of 10 years. Proinflammatory cytokines, such as IL-33, osteopontin (OPN), IL-17, and TNF-α, are involved in both psoriasis and PsA pathogenesis as well as in bone homeostasis. These cytokines induce the release of a wide range of pro-osteoclastogenic factors in the skin, such as RANKL. Furthermore, RANKL serum levels and osteoclast number and activity are influenced by the severity of skin lesions in psoriatic patients with and without arthritis^34^. We previously reported that MHP1 could inhibit RANKL-induced osteoclast differentiation^17^. MHP1-AcN treatment may prove advantageous for psoriatic arthritis, which needs further study.

Taken together, we showed that the systemic administration of the novel, modified partial agonist of RANKL, MHP1, with N-terminal acetylation and C-terminal amidation (MHP1-AcN), could inhibit the development of IMQ-induced psoriatic skin lesions and IL-6, IL-23, and IL-17A production by inhibiting TLR-mediated inflammation. Although the exact molecular mechanisms and appropriate long-term risk-benefit effects need further study, the inhibitory effects of MHP1-AcN on IMQ-induced psoriasis and RANKL-induced osteoclast activation make it a promising agent for psoriatic diseases.

## Methods

### Peptide design and synthesis

Synthetic MHP1-AcN (Ac-LMVYVVKTSIKIPSSHNLMKGGSTKNWSGN-NH_2_) was purchased from ILS, Inc. (Ibaragi, Tsukuba, Japan) and dissolved in double-distilled H_2_O to make a 2 mg/mL solution and stored at 4 °C until use.

### IMQ-induced psoriasis model in mice

All experiments were approved by the Institutional Animal Care and Use Committee of Osaka University (27-020-032) and conducted in accordance with the Osaka University Guidelines, which are based on the National Institutes of Health’s Guide for the Care and Use of Laboratory Animals, and with the ARRIVE guidelines. All procedures were performed under isoflurane anesthesia, and all efforts were made to minimize suffering. Nine-week-old male BALB/c mice, that weighed 22–23 g, were purchased from CLEA Japan, Inc. (Tokyo, Japan). Each mouse received a topical dose of 62.5 mg of commercially available 5% IMQ cream (Aldara; 3 M Pharmaceuticals; Maplewood, MN, USA) on the shaved back and the right ear for 8 or 9 consecutive days. Because our previous study showed the effectiveness of intravenous injection of MHP1-AcN at 75–300 µg/mouse in a stroke model^18^ and others showed the effectiveness and safety of subcutaneous injection of 34^35^ or 25^36^ amino acids of synthetic peptides at 0.3, 1, 3, and 10 µg/mouse^35^ or 150 µg/mouse^36^, MHP1-AcN groups were subcutaneously injected (distant site from IMQ application) with 0.1, 1, 10, 100 µg or 250 µg of MHP1-AcN daily for 8 or 9 days immediately before IMQ application. Considering that the mice weighed 22–23 g, we believe that the influence of body weight was small. As a control for MHP1-AcN treatment, 0.45% saline was injected in a similar manner. The scores of erythema and scaling on the dorsal skin at the site of IMQ application were measured on a four-point scale (0 = none; 1 = slight; 2 = moderate; 3 = marked; and 4 = very marked). The relative increase in dorsal skin thickness was measured using a digital caliper and calculated as the percentage change from the baseline thickness. The thickness of the ear was also measured using a digital caliper. Although each animal experiment was performed once, the effectiveness of MHP1-AcN (100 µg/mouse) was examined three times (Figs 1, 2 and Supplemental Fig. 1). Sixty-eight mice were examined in this study.

### Cell culture

RAW 264.7 cells were obtained from the *RIKEN* Gene Bank (Tsukuba, Japan) and maintained in 5% CO_2_ at 37 °C in Dulbecco’s modified Eagle medium (DMEM; Nacalai Tesque, Kyoto, Japan) supplemented with 10% fetal bovine serum (FBS; Thermo Fisher Scientific, Waltham, MA, USA). Cells (4 × 10^5^ cells) were plated in 96-well plastic culture dishes. After overnight culture, the medium was replaced with DMEM supplemented with 4% FBS. R837 (Imiquimod; InvivoGen, San Diego, CA, USA) and MHP1-AcN were added at the same time to the medium, which was then harvested 24 h after stimulation. The final concentration of R837 was 1 µg/mL, following product information. The concentrations of MHP1-AcN were 0.1, 1, and 10 μg/mL because MHP1-AcN at 1 and 10 μg/mL exerted a significant inhibitory effect on LPS-induced inflammation in microglial cells, as previously reported^18^.

### Enzyme-linked immunosorbent assay (ELISA)

The concentrations of IL-6, IL-23, and IL-17A were measured using the following commercially available ELISA kits: IL-6, Mouse IL-6 Quantikine ELISA Kit (R&D Systems, Minneapolis, MN, USA); IL-23, Mouse IL-23 Quantikine ELISA Kit (R&D systems); and IL-17A, Mouse IL-17A Quantikine ELISA Kit (R&D Systems). The serum concentration of each cytokine was determined in duplicate for each mouse sample. The IL-6 concentration in the macrophage culture medium was also analyzed in duplicate for each experimental condition.

### Real-time reverse transcription polymerase chain reaction (qRT-PCR)

Dorsal skins were collected at 32 h and d9 after IMQ application. mRNA was isolated using an RNeasy Fibrous Tissue Mini Kit (QIAGEN, Germantown, MD, USA) according to the manufacturer’s instructions. cDNA was synthesized using a High-Capacity cDNA Reverse Transcription Kit (Applied Biosystems, Foster City, CA, USA) according to the manufacturer’s instructions. The oligonucleotide primers were purchased according to the following identifications: *Il6*, Mm00446190; *Il23*, Mm00518984; *Il17a*, Mm00439618; and *Gapdh*, Mm99999915 (Applied Biosystems). The 5′ nuclease assay PCRs were performed in a MicroAmp Optical 384-well reaction plate using an ABI PRISM 7900 Sequence Detection System (Applied Biosystems). The expression levels of the target genes were quantified with triplicate repeated wells by comparing the fluorescence generated by each example with that of a serially diluted standard and were then normalized to the level of *Gapdh* expression in each individual sample. We selected the reference gene according to previous studies^2,20^.

### Histological analysis

Mice were perfused with 4% paraformaldehyde (PFA), and the dorsal skin was fixed in 10% neutral-buffered formalin and then processed and embedded in paraffin blocks. Samples were cut into 4-μm-thick sections using a rotary microtome and stained with H&E (Muto Pure Chemicals Co., Ltd.; Tokyo; Japan). Stained sections were observed using a digital microscope (FSX-100; Olympus, Tokyo, Japan). Acanthosis was calculated as the area of epidermis (µm^2^)/the length of the basement membrane (µm) using ImageJ (National Institutes of Health, Bethesda, MD, USA). Inflammatory infiltrate was quantified and shown as the number of infiltrated cells/(0.03 mm^2^).

### Statistical analysis

All values are expressed as the mean ± standard error of the mean (SEM). Multiple comparisons were evaluated by ANOVA, followed by Dunnett’s multiple comparison test. Two groups were compared using an unpaired *t* test. Differences were considered significant at *p* < 0.05. Statistical analyses were performed using the software Prism 6.0 (GraphPad, Inc.; San Diego; CA; USA).

## Supplementary information

Supplementary Info

## Data Availability

The data that support the findings of this study are available from the corresponding author upon reasonable request.
