# Supplementary material for: Preventative effects of the partial RANKL peptide MHP1-AcN in a mouse model of imiquimod-induced psoriasis
Source: Sci Rep. 2019 Oct 28;9:15434. doi: 10.1038/s41598-019-51681-0 (PMC6817907; doi:10.1038/s41598-019-51681-0)

**Preventative effects of the partial RANKL peptide MHP1-AcN in a mouse model of imiquimod-induced psoriasis**

Nan Ju^1,2^, Munehisa Shimamura^1,3*^, Hiroki Hayashi^1^, Yuka Ikeda^5^, Shota Yoshida^1,2^,

Ayumi Nakamura^4^, Ryuichi Morishita^5^, Hiromi Rakugi^2^, Hironori Nakagami^1^*

1. Department of Health Development and Medicine, Osaka University Graduate School of Medicine, Japan
2. Department of Geriatric and General Medicine, Osaka University Graduate School of Medicine, Japan
3. Department of Neurology, Osaka University Graduate School of Medicine, Japan
4. Department of Pharmacy and Department of Medical Innovation, Osaka University Hospital, Japan
5. Department of Clinical Gene Therapy, Osaka University Graduate School of Medicine, Japan

*Corresponding authors: Munehisa Shimamura^1,2^, Hironori Nakagami^1^

Email addresses: shimamuu@cgt.med.osaka-u.ac.jp, nakagami@gts.med.osaka-u.ac.jp

^1^Department of Health Development and Medicine and ^2^Department of Neurology, Osaka University Graduate School of Medicine, Center of Medical Innovation and Translational Research (6th floor, Room 0612B), Osaka University, 2-2 Yamada-oka, Suita, Osaka 565-0871, Japan

**Supplemental Figure**

**Supplemental Figure 1. Higher dose of MHP1-AcN (250 µg/mouse) did not further inhibit the development of IMQ-induced skin lesions nor IL-17A production**

MHP1-AcN (100 or 250 µg/mouse) or saline was systemically administrated by daily subcutaneous injection at a distant site from IMQ application. (A) Dorsal skin erythema and scales were recorded and measured on a four-point scale (0 = none; 1 = slight; 2 = moderate; 3 = marked; and 4 = very marked). Dorsal skin thickness was measured using a digital caliper and calculated as the percentage change from baseline. (B) Right ear thickness from day 0-9. (C) Mice were sacrificed at d9 and serum IL-17A levels were measured by ELISA. **P < 0.05*, ***P* < 0.01 vs. the saline-treated IMQ group. N = 3 per group. All values are expressed as mean with SEM.

**Supplementary Figure 1.**


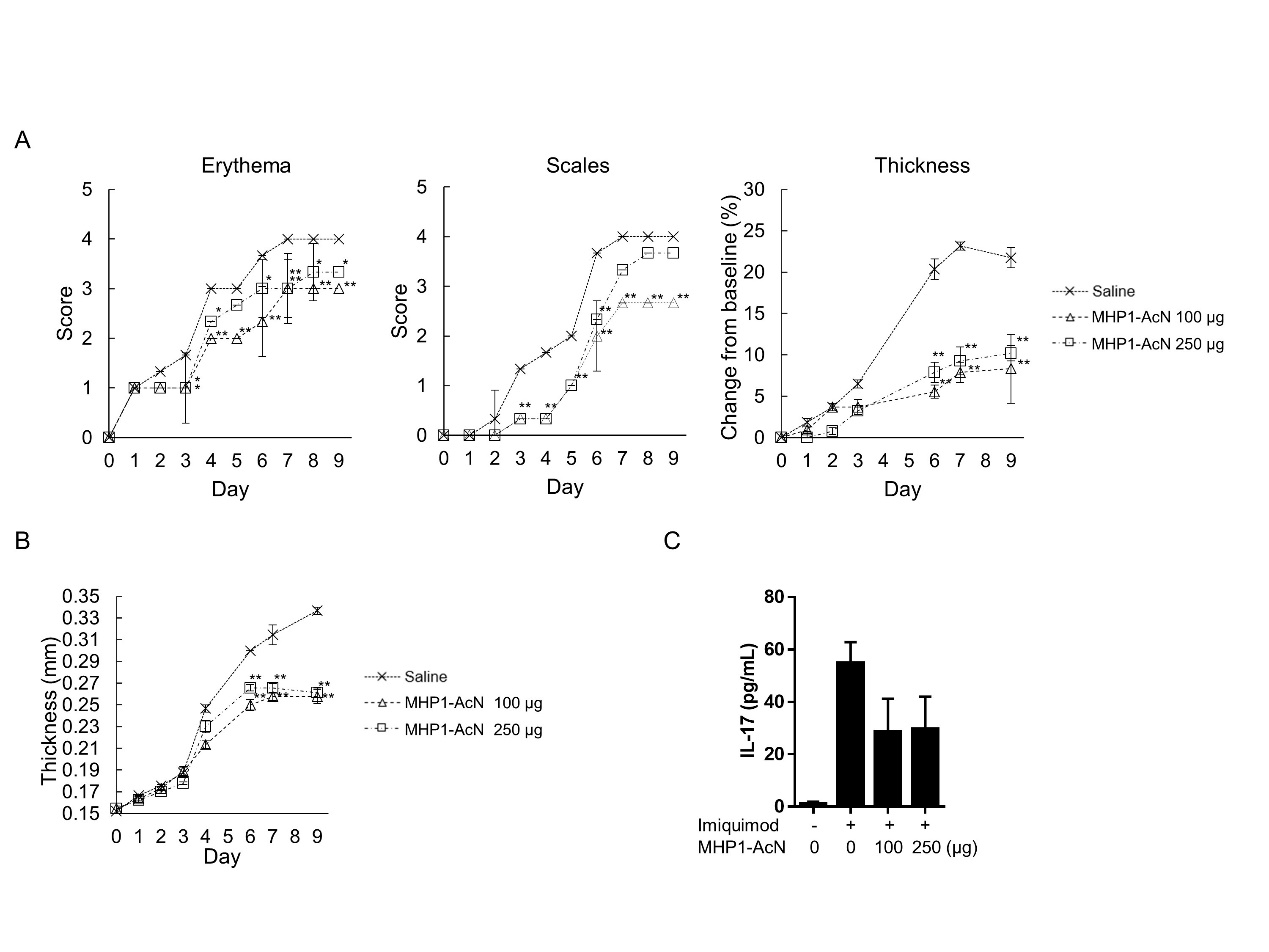

Supplement: Supplementary file 1 — Supplementary Info [file 41598_2019_51681_MOESM1_ESM.docx]
